# Supplementary material for: Cortical Location of Language Function May Differ between Languages While White Matter Pathways Are Similar in Brain Lesion Patients
Source: Brain Sci. 2023 Jul 29;13(8):1141. doi: 10.3390/brainsci13081141 (PMC10452579; doi:10.3390/brainsci13081141)
Supplement: Supplementary file 1 [file brainsci-13-01141-s001.zip › brainsci-2375747-supplementary.pdf]

Stimulation Site	(1) Balto-Slavic and Indo-European Languages		(2) Languages of Bilingual Patients	
	U	*p*	*Z*	*p*
1	213.50	.026**	-1.725	.084*
2	274.50	.531	-.431	.667
3	281.50	.632	-1.579	.114
4	295.00	.891	-1.786	.074*
5	280.00	.630	-1.363	.173
6	296.00	.923	-1.414	.157
7	284.00	.714	-1.754	.079*
8	290.50	.815	-.863	.388
9	282.50	.660	-.312	.755
10	281.00	.624	-1.131	.258
11	271.00	.427	-1.089	.276
12	287.50	.747	-.539	.590
13	273.50	.511	-.632	.527
14	243.50	.201	-.184	.854
15	239.00	.070*	-.447	.655
16	252.50	.221	< .001	1.000
17	211.50	.046**	-.634	.526
18	240.50	.151	-1.511	.131
19	291.50	.822	-.730	.465
20	236.50	.109	-.073	.942
21	251.50	.211	-1.052	.293
22	273.00	.535	< .001	1.000
23	267.50	.450	< .001	1.000
24	241.00	.145	-.447	.655
25	222.00	.027**	-.323	.746
26	246.00	.194	-.677	.498
27	273.50	.482	-1.903	.057*
28	291.50	.846	-.577	.564
29	250.00	.209	-.108	.914

30	262.00	.360	-.682	.495
31	248.00	.206	-.302	.763
32	276.50	.582	-1.786	.074*
33	278.50	.568	-1.069	.285
34	270.00	.410	-1.194	.233
35	252.50	.243	-1.342	.180
36	280.00	.624	-.944	.345
37	286.00	.717	-.272	.785
38	288.50	.772	-1.476	.140
39	229.50	.100*	-2.264	.024**
40	244.00	.137	-1.054	.292
41	260.50	.308	-1.289	.197
42	282.50	.652	-.272	.785
43	272.00	.481	-.552	.581
44	293.00	.866	-1.550	.121
45	268.50	.446	-1.236	.216
46	270.00	.409	-2.414	.016**

Supplementary Table S1: Comparison of Error Rates Between (1) Balto-Slavic and Indo-European Languages and (2) Languages of Bilingual Patients

The table shows the results of the Mann-Whitney U tests comparing the error rates between Balto-Slavic (N = 24) and Indo-European languages (N = 25) and the results of the Wilcoxon Signed Rank Z tests comparing the error rates between the two spoken languages of bilingual patients (N = 18). One asterisk (*) marks differences that were significant at $\alpha = .10$. Two asterisks (**) mark differences that were significant at $\alpha = .05$. After correction for multiple testing, no significant differences were found.

Stimulation Site	Subcortical Pathway Volumes					
	FA = .10			FA = .15		
	IFOF	SLF/AF	FAT	IFOF	SLF/AF	FAT
**1**	.11	-.29	,01	.03	-.29	-.18
**2**	.02	< .01	.04	-.07	-.29	.18
**3**	.19	-.15	-.02	.13	-.37	.22
**4**	.32	.03	.35	.33	.24	.11
**5**	-.22	-.14	-.10	-.25	-.22	-.28
**6**	-.21	-.48*	-.05	-.27	-.60*	-.25
**7**	.03	-.07	.13	-.09	-.13	.15
**8**	-.18	-.03	-.09	-.28	.01	-.01
**9**	.31	.01	.31	.34	.17	.20
**10**	-.29	-.10	-.08	-.47*	-.47*	-.17
**11**	.18	-.02	-.03	.22	.10	-.25
**12**	-.18	-.28	.06	< .01	-.26	.01
**13**	.03	-.19	-.04	-.14	-.25	.03
**14**	.25	.13	.30	.29	.28	.22
**15**	.13	-.09	-.02	-.11	-.23	.06
**16**	.13	-.30	.03	.25	-.07	-.17
**17**	.21	.13	.18	-.01	-.15	.21
**18**	-.33	-.18	-.29	-.45*	-.26	-.32
**19**	-.30	-.43*	-.18	-.35	-.53*	-.38
**20**	-.23	-.13	-.03	-.19	-.41	.03
**21**	.02	-.11	-.04	-.04	-.08	.05
**22**	.24	-.16	-.06	.02	-.30	-.09
**23**	.23	.09	.01	-.05	-.20	< .01
**24**	.21	-.15	.11	.10	-.07	.21
**25**	.21	-.04	.22	.11	-.37	.36
**26**	.02	-.11	.12	.02	-.07	.10
**27**	-.35	-.10	-.05	-.38	-.31	-.22
**28**	-.39	-.12	.07	-.29	-.24	.05
**29**	-.13	-.29	-.19	-.20	-.36	-.18
**30**	.29	.26	.18	.06	.15	.16

**31**	.22	-.02	-.06	.01	-.14	.19
**32**	.24	-.34	.11	.21	-.31	.11
**33**	< .01	.01	.04	-.03	-.23	-.13
**34**	-.02	-.23	.22	.18	-.23	.23
**35**	.26	-.01	.14	.26	.06	.01
**36**	.01	-.25	-.22	-.16	-.21	-.23
**37**	.07	-.06	.10	.04	.04	.09
**38**	.10	-.15	.46*	.26	-.34	.38
**39**	.20	-.11	.26	.15	-.23	.10
**40**	.40	.06	.16	.37	.19	.18
**41**	.54*	.02	.36	.60*	.29	.18
**42**	.13	-.07	-.01	-.12	-.39	-.06
**43**	.18	.17	.31	.23	.16	-.05
**44**	.21	-.22	-.12	.18	-.08	-.03
**45**	-.30	-.06	-.09	-.28	-.16	-.10
**46**	.25	-.28	-.09	.20	-.25	-.05

Supplementary Table S2: Correlations between error rates and relative subcortical pathway volumes in Balto-Slavic languages

The table shows the results of the Spearman's correlations between error rates and relative pathway volumes of the IFOF, SLF/AF and FAT in the Balto-Slavic language group. The asterisk (*) marks correlations that were significant at α = .05. Abbreviations: FA = fractional anisotropy, IFOF = inferior fronto-occipital fascicle, SLF/AF = superior longitudinal fascicle/arcuate fascicle, FAT = frontal aslant tract

**Stimulation Site**	**Subcortical Pathway Volumes**					
	FA = .10			FA = .15		
	IFOF	SLF/AF	FAT	IFOF	SLF/AF	FAT
**1**	.11	-.29	.01	.13	.12	.06
**2**	.02	< .01	.04	-.03	.02	.01
**3**	.19	-.15	-.02	-.09	.16	.31
**4**	.32	.03	.35	-.25	-.02	.31
**5**	-.22	-.14	-.10	.10	.32	.18
**6**	-.21	-.48*	-.05	.22	-.08	.29
**7**	.03	-.07	.13	.32	.45*	.06
**8**	-.18	-.03	-.09	-.30	.13	.24
**9**	.31	.01	.31	.02	.26	.33
**10**	-.29	.10	-.08	.07	.20	.13
**11**	.18	-.02	-.03	.10	-.12	-.03
**12**	-.18	-.28	.06	.34	.18	-.31
**13**	.03	-.19	-.04	.02	.14	-.21
**14**	.25	.13	.30	.08	.37	-.14
**15**	.13	-.09	-.02	-.05	-.23	.20
**16**	.13	-.30	.03	.39	.26	.12
**17**	.21	.13	.18	.37	.07	-.24
**18**	-.33	-.18	-.29	-.01	-.04	.04
**19**	-.30	-.43*	-.18	.03	.13	.35
**20**	-.23	-.13	-.03	.22	.28	-.17
**21**	.02	-.11	-.04	.47*	.41*	-.10
**22**	.24	-.16	-.06	.04	-.06	.18
**23**	.23	.09	.01	.34	.14	.09
**24**	.21	-.15	.11	.21	.17	.42*
**25**	.21	-.04	.22	-.03	.14	.10
**26**	.02	-.11	.12	.19	.10	-.15
**27**	-.35	-.10	-.05	.20	.16	-.09
**28**	-.39	-.12	.07	.29	.08	.24
**29**	-.13	-.29	-.19	.31	-.04	.19
**30**	.29	.26	.18	.10	.25	.22

**31**	.22	-.02	-.06	-.17	.09	.37
**32**	.24	-.34	.11	.25	-.31	.04
**33**	< .01	.01	.04	.19	.18	.15
**34**	-.02	-.23	.22	.47*	.18	-.20
**35**	.26	-.01	.14	.19	.23	-.04
**36**	.01	-.25	-.22	.13	.44*	.45*
**37**	.07	-.06	.10	.22	.11	.02
**38**	.10	-.15	.46*	-.30	.06	.59*
**39**	.20	-.11	.26	.11	.11	.26
**40**	.40	.06	.16	-.30	-.21	< .01
**41**	.54*	.02	.36	.15	.02	.14
**42**	.13	-.07	-.01	-.11	.11	.51*
**43**	.18	.17	.31	< .01	.19	.52*
**44**	.21	-.22	-.12	-.03	-.08	.29
**45**	-.30	-.06	-.09	.07	.11	.47*
**46**	.25	-.28	-.09	-.19	.17	.06

Supplementary Table S3: Correlations between error rates and subcortical pathway volumes in Indo-European languages

The table shows the results of the Spearman's correlations between error rates and relative pathway volumes of the IFOF, SLF/AF and FAT in the Indo-European language group. The asterisk (*) marks correlations that were significant at $\alpha = .05$. Abbreviations: FA = fractional anisotropy, IFOF = inferior fronto-occipital fascicle, SLF/AF = superior longitudinal fascicle/arcuate fascicle, FAT = frontal aslant tract
